# Prévalence, facteurs associés et prédisposant au syndrome métabolique chez les personnes vivants avec le VIH sous traitement antirétroviral à Porto-Novo en 2014

**DOI:** 10.11604/pamj.2015.22.296.7923

**Published:** 2015-11-24

**Authors:** Alassani Adébayo, Dovonou Comlan Albert, Sossou Ericie, Attinsounon Cossi Angelo, Gninkoun Jules, Wanvoegbe Armand, Ahoui Séraphin, Codjo Léopold, Ade Gabriel

**Affiliations:** 1 Centre Hospitalier Universitaire Départemental du Borgou-Alibori, Parakou, Bénin; 2 Faculté des Sciences de la Santé, UAC Cotonou, Bénin; 3 Centre National Hospitalier Universitaire Hubert Koutoucou Maga, Cotonou, Bénin; 4 Centre Hospitalier Universitaire Départemental de l'Ouémé-Plateau, Porto-Novo, Bénin

**Keywords:** Syndrome métabolique, VIH, Porto-Novo, Bénin, Metabolic syndrome, HIV, Porto-Novo, Benin

## Abstract

**Introduction:**

Le syndrome métabolique est associé aux maladies cardiovasculaires. L'infection au VIH est devenue aujourd'hui une maladie chronique. L'objectif de cette étude est de déterminer la prévalence, les facteurs associés et prédisposant au syndrome métabolique chez les patients infectés par le VIH sous traitement antirétroviral.

**Méthodes:**

Il s'est agi d'une étude transversale, descriptive et analytique. La population d’étude est constituée des patients vivant avec le VIH sous antirétroviral suivis au Centre Hospitalier Universitaire de l'Ouémé-Plateau. Le syndrome métabolique a été défini selon les critères de la Fédération Internationale du Diabète.

**Résultats:**

La population était constituée de 244 patients. La prévalence du syndrome métabolique était de 18,03% avec une prédominance féminine (74,6%). La moyenne d’âge était de 40,7 ± 9,71 ans. Les facteurs associés au syndrome métabolique étaient le sexe féminin, la sédentarité, l'antécédent d'HTA, le surpoids, l'apport énergétique élevé, l'apport lipidique élevé, la consommation d'alcool, la consommation de tabac et l'hypercholestérolémie. Les facteurs prédisposant au syndrome métabolique étaient la présence de l'HTA, le tour de taille élevé, l'hyperglycémie, l'hypocholestérolémie HDL et l'hypertriglycéridémie.

**Conclusion:**

Le syndrome métabolique est fréquent chez les patients infectés par le VIH sous traitement antirétroviral. Une prévention prenant en compte les facteurs associés et prédisposant s'avère nécessaire.

## Introduction

L'infection par le VIH demeure l'une des plus grandes préoccupations de santé publique mondiale actuelle. L’évolution de cette épidémie a pris un nouveau visage au cours de ces dernières années. En 2012, on estimait à 35,3 millions le nombre de personnes vivant avec le VIH dans le monde. L'Afrique Sub-saharienne, la région abritant à peine 12% de la population mondiale, était la plus touchée avec 70% de personnes vivant avec le VIH [1]. Depuis l'avènement du traitement antirétroviral hautement actif, on a assisté à une amélioration de la qualité de vie des individus infectés par le VIH; ceci en rapport à la diminution de la fréquence des affections opportunistes [2]. L'infection au VIH est donc devenue une maladie chronique. Elle est de plus en plus caractérisée par la fréquence des anomalies métaboliques en rapport avec la chronicité de l'infection mais aussi les effets secondaires des antirétroviraux [3]. En effet, l'infection au VIH est fortement associée à des anomalies du métabolisme lipidique, notamment une hypercholestérolémie, une élévation des taux du VLDL et des triglycérides et une diminution du taux de cholestérol HDL. On assiste ainsi à une mauvaise répartition corporelle de la graisse et à l'insulinorésistance [4]. Ces différentes anomalies favorisent le syndrome métabolique. Les personnes vivant avec le VIH sous traitement antirétroviral ont un risque élevé de développer le syndrome métabolique comparativement à la population générale [5]. Le syndrome métabolique augmente le risque de maladies cardio-vasculaires et de diabète de type 2 [6, 7]. Ce qui crée chez les patients infectés par le VIH une susceptibilité élevée à ces maladies chroniques. La connaissance des facteurs associés et prédisposant au syndrome métabolique chez les personnes vivant avec le VIH s'avère indispensable pour une meilleure prévention. L'objectif de cette étude était de déterminer la prévalence du syndrome métabolique ainsi que les facteurs associés chez les personnes vivant avec le VIH sous traitement antirétroviral et suivies au CHUD-OP à Porto-Novo.

## Méthodes

Il s'est agi d'une étude transversale, descriptive et analytique. La population d’étude était constituée des personnes vivant avec le VIH sous antirétroviral suivis au Centre Hospitalier Universitaire Départemental de l'Ouémé-Plateau. Le syndrome métabolique a été défini selon les critères de la Fédération Internationale du Diabète. Ces critères sont les suivants: un périmètre abdominal ≥ 94 cm chez l'homme et ≥ 80 cm chez la femme, c'est le critère principal; une pression artérielle ≥130/85 mmHg ou traitement spécifique de l'hypertension artérielle, une glycémie à jeun ≥1 g/L ou traitement spécifique, une triglycéridémie à jeun ≥1,50 g/L ou traitement spécifique et une cholestérolémie HDL < 0,40 g/L chez l'homme et < 0,50 g/L chez la femme ou traitement spécifique. La présence du critère obligatoire associée à deux autres critères définissent le syndrome métabolique. Les patients ayant un indice de masse corporelle (IMC) ≥25 kg/m^2^ sont considérés comme en surpoids. Le bilan lipidique et la glycémie ont été réalisés chez les patients à jeun le jour de la collecte des données. Les patients hospitalisés, les femmes enceintes ou tout autre patient ayant une pathologie favorisant une augmentation du volume de l'abdomen ont été exclus de l’étude. Une cholestérolémie totale supérieure à 2 g/L est élevée; une cholestérolémie LDL supérieure à 1 g/L est élevée, une cholestérolémie HDL inférieure à 0,5 g/L chez les femmes et inférieure à 0,4 g/L chez l'homme est basse, une triglycéridémie supérieure à 1,5 g/L est élevée. Une glycémie supérieure ou égale à 1,1 g/L a été considérée comme hyperglycémie. L'apport énergétique a été évalué par le rappel des 72 heures. Les besoins énergétiques ont été calculés par le logiciel Alimenthèque en fonction du poids corporel, de la taille et du niveau d'activité physique de chaque patient. Les besoins énergétiques ont été majorés de 10% pour satisfaire à l'excédent calorique nécessaire chez les patients vivant avec le VIH asymptomatiques. L'apport en énergie a été considéré comme normal si elle est égale à +/- 200 kcal des besoins énergétiques. Les apports en lipides ont été considérés comme normaux s'ils sont inférieurs à 30% des besoins énergétiques. L'analyse des données a été faite par le logiciel Statistical Package for Social Sciences (SPSS) version 20.0.

## Résultats

Parmi les 244 patients qui ont été inclus dans l’étude, on a noté une prédominance des femmes (74,6%). La moyenne d’âge était de 40,7 ± 9,71 ans avec une prédominance des patients âgés de moins de 40 ans (57,4%). Le schéma d'antirétroviral (ARV) utilisé était dominé par l'association de deux inhibiteurs de la transcriptase inverse (INTI) et d'un inhibiteur non nucléotidique de transcriptase inverse (INNTI) chez 239 patients (97,95%). Le reste des patients était sous association de deux INTI et d'une anti-protéase (IP). La prévalence du syndrome métabolique était de 18,44% (45 patients). La prévalence de l'hypertension artérielle (HTA) était de 24,6% (60 patients). La prévalence de diabète était de 2,04% (5 patients). L'hyperglycémie est observée chez 26 patients (10,66%). Près de la moitié des patients (49,6%) était sédentaire. Le surpoids était observé chez 75 patients (30,7%). Le tour de taille était élevé chez 51,22% (125 patients). La moyenne de la cholestérolémie totale était de 1,90 ± 0,37 g/L, celle de la cholestérolémie LDL 1,06 ± 0,31 g/L, celle de la cholestérolémie HDL 0,66 g/L ± 0,14 et celle de la triglycéridémie 0,85 ± 0,25 g/L. L'hypercholestérolémie totale était observée chez 101 patients (41,39%), l'hypercholestérolémie LDL chez 84 patients (34,83%), l'hypocholestérolémie HDL chez 68 patients (27,87%) et l'hypertriglycéridémie chez 44 patients (18,03%). L'apport énergétique était élevé chez 63 patients (25,8%), celui lipidique était élevé chez 142 patients (58,2%). Les facteurs associés au syndrome métabolique étaient le sexe féminin, la sédentarité, l'antécédent d'HTA, le surpoids, l'apport énergétique élevé, l'apport lipidique élevé, la consommation d'alcool, la consommation de tabac et l'hypercholestérolémie LDL (Tableau 1 et Tableau 2). Les facteurs prédisposant au syndrome métabolique étaient la présence de l'HTA, le tour de taille élevé, l'hyperglycémie, l'hypocholestérolémie HDL et l'hypertriglycéridémie (Tableau 3).

**Tableau 1:** Facteurs associés au syndrome métabolique chez les PVVIH sous ARV au CHUD-O/P en 2012

Paramètres	Syndrome métabolique		
	Présence(n/%)	Absence(n/%)	p-value
Age < 40 ans	23(16,4)	117(83,6)	0,449
Age = 40 ans	21(20,2)	83(79,8)	
Féminin	39(20,6)	143(79,4)	0,018
Masculin	5(10,9)	57(89,1)	
Antécédents d'HTA	12(15,0)	68(85,0)	0,0287
Pas d'antécédents d'HTA	32(9,5)	132(80,5)	
Antécédents de diabète	1(15,0)	29(85,0)	0,39
Pas d'Antécédents de diabète	33(15,4)	181(84,6)	
IMC < 25 kg/m^2^	69(40,8)	100(59,2)	0,00019
IMC < 25 kg/m^2^	50(66,7)	25(33,3)	
Moins d'un an d'ARV	0(0,0)	6(100,0)	0,244
2INTI + INNTI	44(18,5)	194(81,5)	
Un an ou plus d'ARV	43(18,0)	196(82,0)	0,50
2INTI + IP	1(20,0)	4(80,0)	

**Tableau 2:** Facteurs associés au syndrome métabolique chez les PVVIH sous ARV au CHUD-O/P en 2012

Paramètres	Syndrome métabolique		
	Présence (n/%)	Absence (n/%)	p-value
Apport énergétique bas ou normal	24(13,3)	157(86,7)	0,001
Apport énergétique élevé	20(31,8)	43(68,3)	
Apport lipidique normal	7(6,9)	95(93,1)	0,014
Apport lipidique élevé	25(17,6)	117(82,4)	
Pas de consommation alcool	9(6,8)	123(93,2)	0,000001
Consommation alcool	35(31,3)	77(68,8)	
Consommation tabac	43(17,7)	200(82,3)	0,018
Pas de consommation tabac	1(100,0)	0(0,0)	
Actif	13(10,6)	110(89,4)	0,025
Sédentaire	31(21,6)	90(74,4)	
Hypercholestérolémie totale	13(12,9)	90(87,1)	0,76
Pas d'hypercholestérolémie totale	31(21,7)	112(78,3)	
Hypercholestérolémie LDL	21(25,0)	63(75,0)	0,03
Pas d'hypercholestérolémie LDL	13(8,1)	147(91,9)	

**Tableau 3:** Facteurs prédisposant au syndrome métabolique chez les PVVIH sous ARV au CHUD-O/P en 2012

Paramètres	Syndrome métabolique				
	Présence		Absence		p-value
	Effectif	%	Effectif	%	
**Tour de taille**					
Homme					0,008
< 94cm	1	2,6	38	97,4	
≥ 94cm	6	24,0	19	76,0	
Femme					0,000
< 80cm	1	1,2	79	98,8	
≥ 80cm	36	36,0	64	64,0	
**HTA**					0,001
Présence	15	25,0	45	60,0	
Absence	29	15,8	155	84,2	
**Hyperglycémie**					0,037
Présence	15	57,7	11	42,3	
Absence	29	13,3	189	86,7	
**Diabète**					0,121
Présence	1	20,0	4	80,0	
Absence	43	18,0	196	82,0	
**Hypertriglycéridémie**					0,028
Présence	10	22,7	34	77,3	
Absence	34	17,0	166	83,0	
**Hypocholestérolémie HDL**					0,01
Présence	30	44,1	38	55,9	
Absence	14	7,9	162	92,0	

## Discussion

La présente étude s'est intéressée au syndrome métabolique chez les patients infectés par le VIH sous antirétroviraux. Plusieurs critères permettent de définir le syndrome métabolique. Le critère utilisé qui était celui de la Fédération Internationale du Diabète a été acceptable pour apprécier le syndrome métabolique. La population d’étude était jeune avec une moyenne d’âge de 40,7 ± 9,71 ans. Cette jeunesse de la population des patients infectés par le VIH est aussi soulignée par Malangu et al. et Alencastro et al. qui rapportèrent respectivement une moyenne d’âge de 40,5 ans et 38,6 ans [5, 8]. C'est donc une infection touchant avec prédilection des adultes jeunes ce qui a un impact économique surtout dans les pays en voie de développement. Les patients inclus dans l’étude étaient majoritairement les femmes (3 femmes pour 1 homme). Cette prédominance féminine était aussi observée dans l’étude de Mbunkah et al. [6]. La prévalence du syndrome métabolique observée dans l’étude était de 18,44% soit 2 patients sur 10. Tesfaye et al. ont rapporté une prévalence du syndrome métabolique de 23,8% proche de la notre avec les mêmes critères d’évaluation [9]. Les facteurs associés au syndrome métabolique trouvés dans la présente étude étaient le sexe féminin, la sédentarité, l'antécédent d'HTA, le surpoids, l'apport énergétique élevé, l'apport lipidique élevé, la consommation d'alcool, la consommation de tabac et l'hypercholestérolémie LDL. Plusieurs auteurs ont rapporté des résultats similaires. C'est le cas d'Alvarez et al. et Wu et al. pour ce qui est de l'association entre le sexe féminin et le syndrome métabolique [10, 11]. La sédentarité était associée au syndrome métabolique dans les études d'Alencastro et al. et de Kagaruki et al. [8, 12].

Le manque d'activité physique favorise une balance énergétique positive avec accumulation de graisse. L'association entre antécédent d'HTA et syndrome métabolique est aussi observée dans l’étude de Wu et al. [11]. Le surpoids a été un facteur favorisant du syndrome métabolique dans les études de Dimodi et al. et de Krishman et al. [2, 13]. Les patients infectés par le VIH ayant un apport énergétique élevé avaient une prévalence significativement élevée du syndrome métabolique selon les résultats rapportés par Tiozzo et al. [14]. Il en est de même de l'apport lipidique élevé qui peut contribuer à l'apport énergétique élevé et à l'accumulation corporelle de graisse favorisant la lipodystrophie. Cette mauvaise répartition des graisses est un facteur favorisant le syndrome métabolique [2, 3]. La consommation de tabac a été associée au syndrome métabolique dans l’étude d'Alencastro et al. [8]. L'alcool est un aliment riche en énergie et peut contribuer à un apport énergétique élevé qui est facteur de risque du syndrome métabolique [15, 16]. En dehors de l'apport énergétique, l'alcool favorise une hypertriglycéridémie qui est une des composantes du syndrome métabolique [17].Dans les études d'Alvarez et al. et Wu et al., l'hypercholestérolémie LDL était associée au syndrome métabolique [10, 11]. L’âge des patients, la durée et le type de traitement antirétroviral n’étaient pas associés au syndrome métabolique dans la présente étude. Dans la littérature les opinions sont divergeantes. Pour Malangu et al. et Tiozzo et al., l’âge n’était pas associé au syndrome métabolique [5, 14] tandis que Elgalib et al., et Mbunkah et al. avaient rapporté que les sujets plus âgés sont plus à risque de développer le syndrome métabolique [3, 6]. Pour ce qui est de la durée du traitement antirétroviral, Tesfaye n'avait pas observé une relation avec le syndrome métabolique [9] contrairement aux résultats rapportés par Wu et al. [11]. L'utilisation d'un inhibiteur de la protéase a été associée au syndrome métabolique dans les études de Malangu et al. et Tiozzo et al. [5, 14]. Mbunkah et al. n'avaient pas noté une relation entre les inhibiteurs de la protéase et le syndrome métabolique [6]. L'un des objectifs de la présente étude était d'identifier les facteurs prédisposant au syndrome métabolique. Toutes les composantes du syndrome métaboliques ont été des facteurs prédisposant au syndrome métabolique chez les patients infectés par le VIH sous antirétroviraux. Les patients ayant un tour de taille élevé étaient prédisposés au syndrome métabolique dans les études de Dimodi et al. et Alvarez et al. [2, 10]. Wu et al. et Tiozzo et al. ont souligné l'association entre l'hypertension artérielle et le syndrome métabolique [11, 14]. L'hyperglycémie était un facteur prédisposant au syndrome métabolique selon les résultats de l’étude de Mbunkah et al. [6]. La prédisposition de l'hypertriglycéridémie et de l'hypocholestérolémie HDL au syndrome métabolique a été rapportée par Kagaruki et al. [12].

## Conclusion

Le syndrome métabolique est une réalité chez les patients infectés par le VIH sous antirétroviraux; sa prévalence était de 18,44% au CHUD-OP à Porto-Novo. Les facteurs associés au syndrome métabolique étaient le sexe féminin, la sédentarité, l'antécédent d'HTA, le surpoids, l'apport énergétique élevé, l'apport lipidique élevé, la consommation d'alcool, la consommation de tabac et l'hypercholestérolémie LDL. Les facteurs prédisposant au syndrome métabolique étaient l'hypertension artérielle, le tour de taille élevé, l'hyperglycémie, l'hypocholestérolémie HDL et l'hypertriglycéridémie. Les mesures de lutte contre les facteurs associés et prédisposant permettront de réduire la prévalence du syndrome métabolique.
